# The Functional Study of Response Regulator ArlR Mutants in *Staphylococcus Aureus*

**DOI:** 10.1007/s12010-024-04919-1

**Published:** 2024-03-26

**Authors:** Jinhong Zhou, Moath Refat, Yucheng Guo, Jiaxin Zhang, Min Jiao, Wenbo He, Xiaoyu He, Mai A. Rabie, Zhenlin Ouyang, Fang Zheng

**Affiliations:** 1 The Key Laboratory of Environment and Genes Related to Disease of Ministry of Education, Health Science Center, Xi’an Jiaotong University, Xi’an, 710061 China; 2 Talent Highland, The First Affiliated Hospital, Xi’an Jiaotong University, Xi’an, 710061 China

**Keywords:** *Staphylococcus aureus*, Response regulator ArlR, Biofilm formation, Adherence

## Abstract

*Staphylococcus aureus* is a major cause of hospital-associated infections worldwide. The organism’s ability to form biofilms has led to resistance against current treatment options such as beta-lactams, glycopeptides, and daptomycin. The ArlRS two-component system is a crucial regulatory system necessary for *S. aureus* autolysis, biofilm formation, capsule synthesis, and virulence. This study aims to investigate the role of the arlR deletion mutant in the detection and activation of *S. aureus*. We created an *arlR* deleted mutant and complementary strains and characterized their impact on the strains using partial growth measurement. The quantitative real-time PCR was performed to determine the expression of *icaA,* and the microscopic images of adherent cells were captured at the optical density of 600 to determine the primary bacterial adhesion. The biofilm formation assay was utilized to investigate the number of adherent cells using crystal violet staining. Eventually, the Triton X-100 autolysis assay was used to determine the influence of *arlR* on the cell autolytic activities. Our findings indicate that the deletion of *arlR* reduced the transcriptional expression of *icaA* but not *icaR* in the *ica* operon, leading to decrease in polysaccharide intercellular adhesin (PIA) synthesis. Compared to the wild-type and the complementary mutants, the *arlR* mutant exhibited decreased in biofilm production but increased autolysis. It concluded that the *S. aureus* response regulatory *ArlR* influences biofilm formation, agglutination, and autolysis. This work has significantly expanded our knowledge of the *ArlRS* two-component regulatory system and could aid in the development of novel antimicrobial strategies against *S. aureus*.

## Introduction

*Staphylococcus aureus*, also known as golden staph, is a gram-positive bacterium categorized as one of the ESKAPE pathogens, the highly virulent antibiotic-resistant bacterial strains, resulting in significant morbidity and mortality rates worldwide [1]. *S. aureus* can cause a variety of infections ranging from mild to severe, including blood septicemia, thrombophlebitis, infectious endocarditis [2–4] osteomyelitis, or device-related infections (DRI) [5], such as intravascular catheters, prosthetic joints, vascular grafts, and pacemakers [6].

*Staphylococcus aureus* has emerged as a leading cause of hospital-associated infections and the most life-threatening multidrug-resistant opportunistic pathogens worldwide [7, 8]. *Methicillin-resistant S. aureus* (MRSA), also known as oxacillin-resistant *S. aureus*, is the most virulent form of *S. aureus*, which has reached a particular epidemic and healthcare threat globally [9, 10]. MRSA is defined as an oxacillin minimum inhibitory concentration (MIC) greater than or equal to 4 mg/L [11]. MRSA has developed resistance to current treatment options such as -lactams, glycopeptides, and daptomycin due to the organism’s capacity to produce biofilms [12, 13], commonly known as the two-component regulatory system (TCS), which functions as a sensory system for *S. aureus* [14]. The success of *S. aureus* in invading humans highly depends on various virulence factors, including exoenzymes, toxins, and adhesions that make the bacterial adhere to the mucous membranes or invade the immune system and adapt to dynamic environmental stimuli to survive [15, 16]. Multiple virulence factors are controlled by the two-component regulatory system (TCS). Most of the *S. aureus* strains possess 16 TCSs, one of which is vital for bacterial viability [17, 18].

The ArlRS TCS has been involved in various pathogenic processes, such as autolysis, biofilm formation, virulence, and capsule synthesis, which plays a vital role in the regulation of clumping and adherence [19, 20]. ArlRS consists of a membrane-bound histidine kinase ArlS that responds to different environmental signals, auto-phosphorylates a conserved histidine residue, and a cognate response regulator ArlR which its aspartate residue receives the phosphate and affects the transcription of a subset of target genes [21, 22]. The *arlR* was found to activate the production of MgrA responsible to controls the virulence factors and the intracellular adhesion gene cluster (*ica* operon) [23, 24]. Additionally, it serves as a direct activator for abcA, a gene that encodes an ATP-dependent transporter associated with cell wall autolysis and -lactam antibiotics resistance [25]. ArlR-MgrA directly controls the cascade that represses the biofilm formation regulator Rbf and upregulates SarX, a Sar transcriptional regulatory protein family member that regulates *ica* expression [26]. It has been reported that MgrA knockout in USA300 resulted in just a slight reduction of MIC for oxacillin (from 64 to 32 mg/L), but arlRS knockout in USA300 results in a significantly decreased MIC value (4 mg/L) [10]. Moreover, the *arlR* mutant has an essential clumping defect for staphylococcal infection. Polysaccharide intercellular adhesion (PIA) is a common element of staphylococcal biofilm [27, 28]. The ArlR plays a vital role in regulating biofilm formation, which can directly bind to the promoter region between *icaR* and *icaADBC*, enhancing the expression of *icaA* and PIA production [29].

The synthesis of PIA is performed via the *ica* locus, which has an operon composed of four open frames named *icaA*,* icaD*, *icaB*, and* icaC*. The *icaR* is likewise a component of the *ica* operon and is located upstream but in the opposite location [30, 31]. Our previous study showed structural insights into the activation and recognition of ArlR, and ArlR recognizes a 20 bp imperfect inverted repeat sequence located at the *ica* operon [32]. Nonetheless, the downstream gene regulatory mechanisms of *arlR* in *S. aureus* are poorly studied. In this research, we have introduced a novel approach to investigate the role of the arlR deletion mutant in detecting and activating *S. aureus*. We have created the strains mutant and complementary mutant, evaluated the effects of the *arlR* in the growth measurement, determined the influence of *arlR* on the cell autolytic activities, and assessed the intracellular adhesion cells agglutination. This study provides an in-depth understanding of the ArlR response regulator and its functions, offering unique opportunities to combat antibiotic resistance and directly target pathogen virulence. The outcomes of this study may also pave the way for further research into targeting the ArlR regulatory system as a novel target for fighting *S. aureus*.

## Materials and Methods

### Bacterial Strains and Primers

The bacterial strains used in this study are listed in Table 1. *S.aureus* were cultured in Trypticase soy broth (TSB) (Oxoid) at 37 °C. *Escherichia coli* cells were cultured in Luria-Bertani (LB) (Oxoid) medium. Cloning of *S. aureus* chromosomal DNA fragments was performed in *Escherichia coli* stains DH5α using plasmid PKOR_1_ and vector pLi50 [33].

**Table 1:** Strains and primers

Strains and primers	Description	Source
*S. aureus * NCTC8325	NCTC 8325 = AS 1.2132	NCTC
*S. aureus* RN4220	Restriction deficient cloning host	ATCC
*E.coli.* DH5α	Host for plasmid construction	Thermo Scientific™
attB1-UP-F	GGGGACAAGTTTGTACAAAAAAGCAGGCTGCAAAGACAATATGATACTTAC	
UP-overlap-R	GCGCAATTTACGTTTTGTTGTACACCTCATATTACGAC	
Down-overlap-F	GTCGTAATATGAGGTGTACAACAAAACGTAAATTGCGC	
attB2-Down-R	GGGGACCACTTTGTACAAGAAAGCTGGGTAGGATGCAATTGTTTTAATG	
pArlR-Kpn1-F	GGGGTACCATATTGCGGTAAGGCCTTGTGTTACAG	
ArlR-HindIII-R	CCCAAGCTTTCATCGTATCACATACCCAACGC	

### Construction of the arlR Mutant Strains

A 1000 bp upstream and downstream region of the *arlR* were amplified by homologous recombinant form *S. aureus* genome DNA using BIO-RAD C1000 Touch PCR thermal cycler, ligated to temperature-sensitive shuttle plasmid PKOR1 through fusion PCR and Gateway cloning technology using primers. The recombinant plasmid was extracted and transformed into a restriction system-defective *S. aureus* RN4220 by electroporation. The target strain was electrophoresed with a modified plasmid. Positive colonies were broth incubated overnight at 30 °C with 2 mL TSB containing 10 µg/mL chloramphenicol (Sigma). The strains were diluted by a ratio of 1:100 into a total volume of 50 mL TSB containing 10 µg/mL chloramphenicol and incubated overnight at 42 °C. The cultivation process was repeated twice. The cultures were diluted before plating 100 µL on TSB plate (10 µg/mL chloramphenicol) by incubating overnight at 42 °C. A single colony was resuspended with 2 mL TSB and incubated overnight at 30 °C without any additive antibiotics to facilitate plasmid excision. This culture’s 100 folded serial dilutions were spread on the TSB containing 1 µg/mL tetracycline (Sigma) and incubated for 24 h at 37 °C. The transformants were screened for chloramphenicol-sensitive colonies, which grown on the TSB plates not on TSB plate containing 10 µg/mL tetracycline. A single colony was isolated and re-cultured on the TSB plates and then inoculated into 5 mL TSB and incubated overnight with continuous shaking at 37 °C. The deleted mutants were identified by PCR using the primers attB_1_-UP-F and attB_2_-Down-R.

### Creation of the arlR Complement

The *arlR* gene pair and its native promoter were amplified with the primer pairs of p*arlR*-Kpn1-F and p*arlR*-HindIII-R using *S. aureus* genome DNA. The PCR product was gel-purified and digested by KpnI and HindIII at 37 °C overnight. The digested fragment was cloned into vector pLi50 and then introduced into *E.coli* DH5α to generate the plasmid pLi50-*arlR*. The restriction defective RN4220 was electroporated by plasmid after genetic modification in RN4220. The recombinant plasmid was transduced into the *S. aureus* △*arlR*.

### Quantitative PCR

For qPCR experiments, *arlR* deleted mutant and complementary strains were activated using 5 mL TSB and incubating overnight at 37 °C. Following incubation, 50 µL cultures were transferred to 50 mL TSB and incubated at 37 °C for 12 h then lysed for 30 min at 37 °C with 100 µL lysostaphin (Sigma). The total RNA was extracted by Qiagen RNeasy Mini kit, and 300 ng of RNA was verified by the A260/A280 ratio of 2.0–2.1. Then the high-capacity cDNA reverse transcription kit (Bio-Rad) was used for the first-strand cDNA synthesis. The qPCR experiments were performed using the Power SYBR Green PCR Master Mix (Bio-Rad) to determine the expression of *icaA* via Bio-Rad CFX Connect Real-Time PCR Detection System by following conditions: 95 °C for 10 min, 95 °C for 10 s, 60 °C for 15 s, and 72 °C for 20 s with 39 cycles. All qRT-PCR primers used for the qRT-PCR (Table 2) were verified using normal PCR. 16 S-RNA was used as a reference gene to standardize all the data.

**Table 2:** qRT-PCR primers

Primer	Sequence
16 S rRNA-F:	GCTGCAGCTAACGCATTAAGCACT
16 S rRNA-R:	TTAAACCACATGCTCCACCGCTTG
*icaR*-F	TCCAGAAAATTCCTCAGGCGT
*icaR*-R	TCAGAGAAGGGGTATGACGGT
*icaADBC*-F	CAGAGGTAAAGCCAACGCAC
*icaADBC*-R	ATCTCACGCGTTGCTTCCAA

### Growth Curves of arlR Wild-Type, Delete Mutant, and Complementation Strains

The strains were incubated overnight with 5 mL of TSB at 37 °C before being diluted 1:100. Using the TSB medium to adjust an OD_600_ to 0.15, cultures were grown at 37 °C with 200 rpm of continuous shaking. The optical density was determined by measuring the OD_600_ every 30 min for 10 h.

### Scanning Electron Microscopy (SEM) Images

The bacterial cultures were grown in 5 mL TSB at 37 °C overnight and then were diluted 1:100 with TSB in 24-well plates (Beyotime, China). After incubation at 37 °C for 24 h without shaking, the catheter fragments were washed for 5 min with PBS three times. The strains were then fixed for 20 min with 4% paraformaldehyde (Sigma), followed by a secondary immobilization with 2.5% glutaraldehyde (Sigma). After overnight incubation, samples were washed three times with phosphate buffers and dehydrated in 30%, 50%, 70%, 80%, 90%, 95%, or 100% ethanol (Sigma) for at least 10 min each. Eventually, the samples were dried, ion-sputter gilded, and then captured using an FEI Quanta FEG 250 SEM with a magnification of x5000 and x20000 times [34].

### Adherence of arlR Delete Mutant and Complementation

The bacterial primary adhesion ability determination was used as previously described in the literature [35] after being modified to meet our study conditions. The overnight-grown strains were inoculated in TSB to the early stationary phase and were centrifuged at 4000 rpm. The bacterial suspensions were adjusted with PBS to an optical density at OD_600_ of 0.1, and 5 mL of each suspension was put on 6-well plates (Beyotime, China) and incubated at 37 °C for 30 min. Each plate was washed five times with PBS, and microscope images of adherent cells were captured (1000 folds magnification).

### Biofilm Formation Assay

The overnight-grown bacterial cultures were adjusted 1:200 in TSB, transferred to 96-well polymer plates (Beyotime, China), and incubated for 24 h at 37 °C. The Petri dishes were gently washed thrice with PBS, fixed for 15 min with 200 µL of 99% methyl alcohol, and dried. The plates were stained with 2% crystal violet for 5 min before being measured at an OD_570_ wavelength.

### Triton X-100-Induced Autolysis Assays

The strain’s autolysis assays were performed as previously described in the literature [28]. Briefly, overnight-grown bacterial cultures were diluted 1:100 in TSB containing 1 M of NaCl (Sigma) and cultivated at 37 °C with continuous shaking until OD_580_ reached approximately 0.7. The cells were centrifuged at 13,800 g at 4 °C for 10 min and washed twice with 50 mL ice-cold water. Then, the cells were resuspended in the same volume containing 50 mM Tris-HCl (PH 7.5) and 0.1% Triton X-100 (Sigma). Bacterial strains were incubated at 30 °C with continuous shaking for 3–5 h, while the OD_580_ was measured every 30 min.

## Results

### Construction of an arlR Delete Mutant Strain of *S. aureus*

**Fig. 1:**
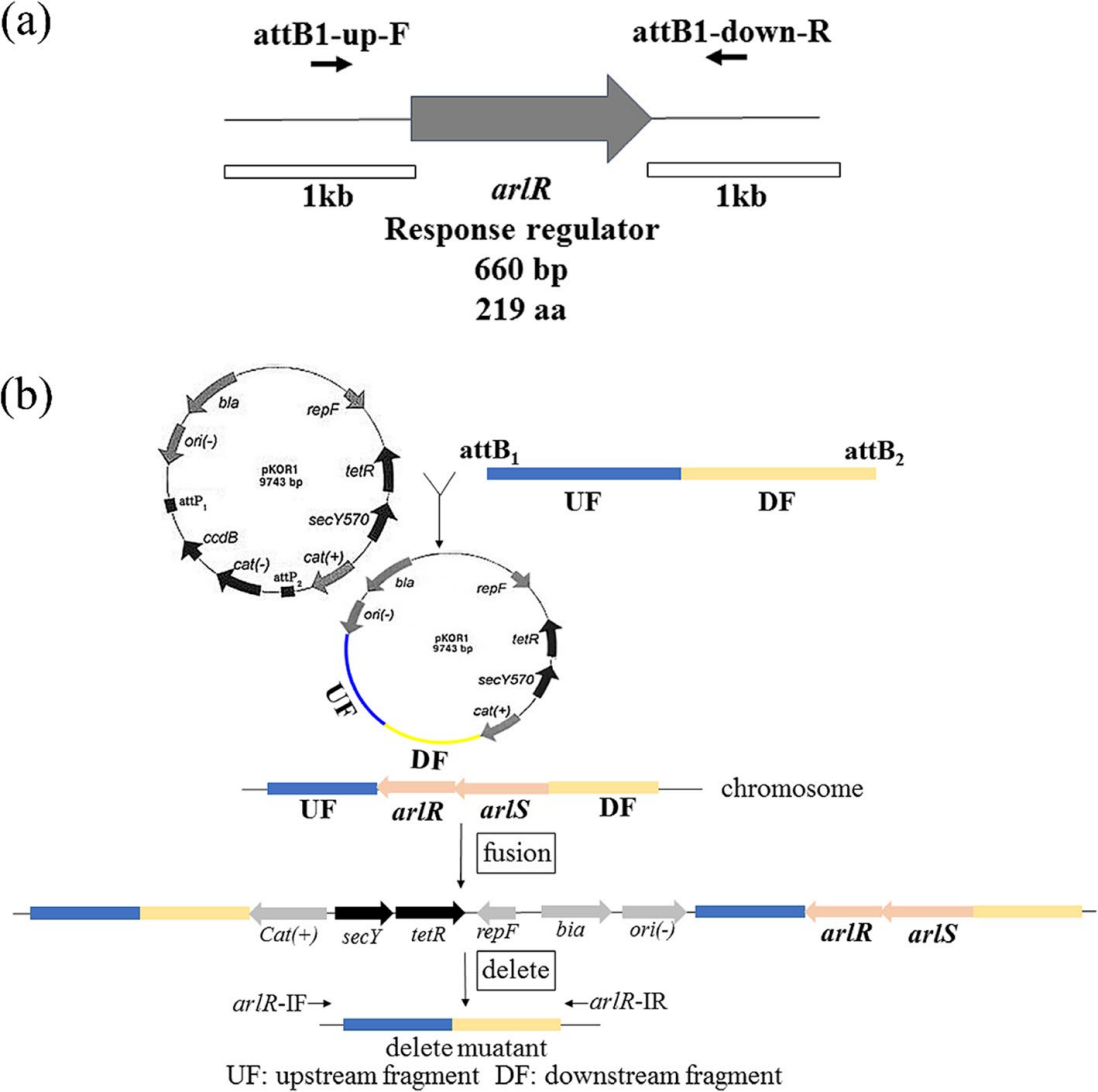
PCR validation of PKOR_1_ allelic replacement. **a**  The primers (attB1-up-F and attB2–down-R) are shown as short black arrows. *arlR* is indicated by an arrow box, while the PCR products used for deletion of *arlR* were shown as boxes with 1 kb size. **b**  Schematic chart for the construction of the *arlR* deleted mutant

A previous study showed the influence of the locus on autolysis, adhesion, and extracellular proteolytic activity in *arlS* mutants [19]. An arlR deleted mutant was created to investigate virulence gene regulation, in which the gene of *arlR* (NZ_CP015758) was deleted. The mutation (△*arlR*) leads to the deletion of 219 amino acids from the predicted protein (Fig. 1a). The experimental procedures were performed as follows (Fig. 1b).

### The Growth Curve of Strains

**Fig. 2:**
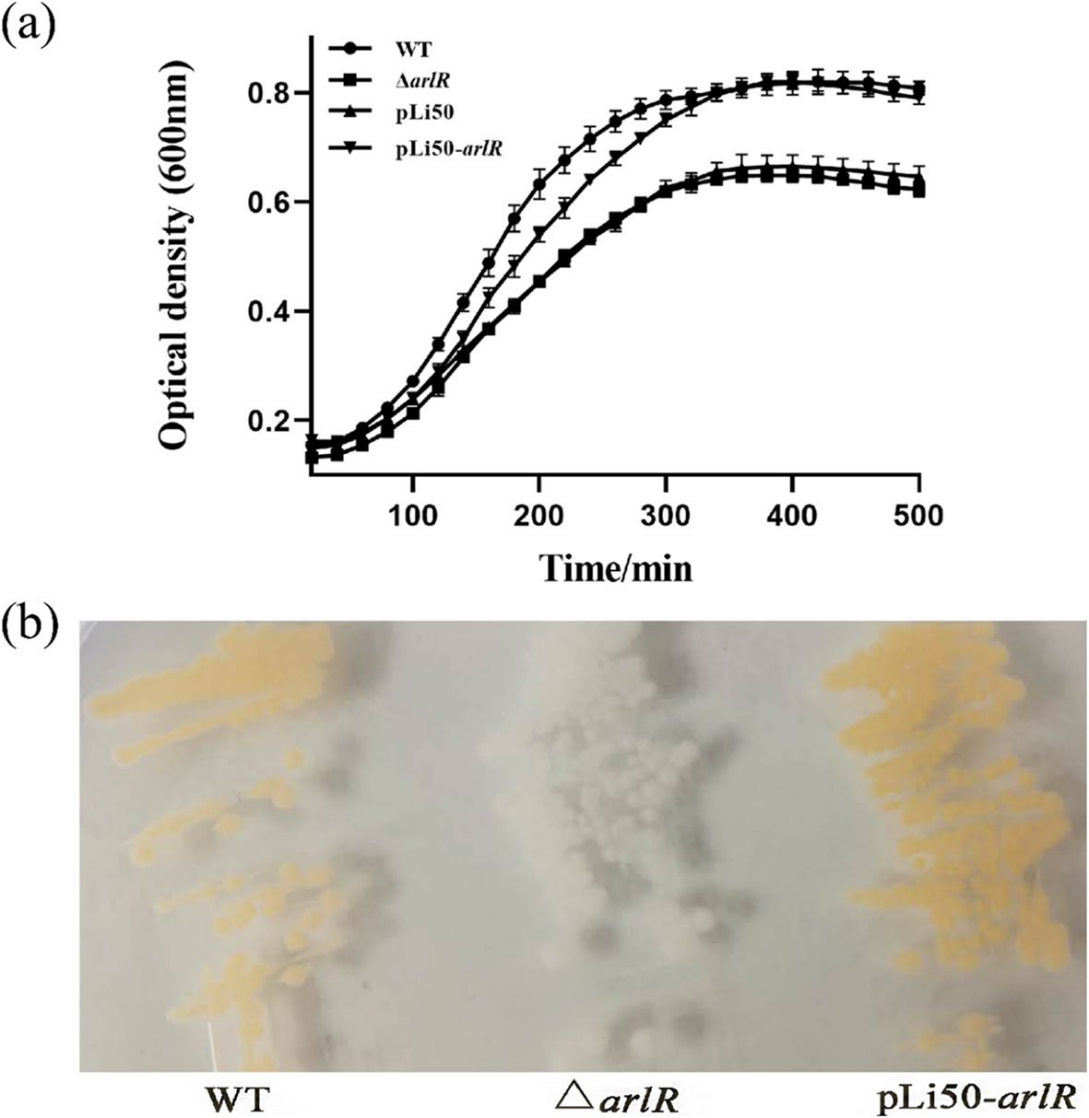
Effects of arlR knockout on *S. aureus* growth. **a**  Growth curves of *S. aureus* strains. Overnight cultures were diluted to OD_600_ = 0.15 as the start point by TSB and incubated at 37 °C with shaking. The value of OD_600_ was measured every 30 min for about 8 h. The experiment was repeated thrice, and the representative data was shown. **b**  Bacterial morphology. Overnight cultures were diluted to 1:10000 with fresh TSB after incubating on TSB plates at 37 °C for 24 h

The *arlR* mutant strain was used to study the influence of *arlR* mutation on the growth rate, autolysis, and biofilm formation. The growth curve of the strains was cultured under the same condition and measured using a spectrophotometer at an optical density of 600 (OD_600_). Both the pLi50 and the *arlR* mutant adopted comparable growth rate patterns. Contrary, the growth rate behavior of the complementary strain was somehow similar to the wild-type strain; however, it was obvious that the wild-type strain’s growth rate was rapidly rising during the mid-exponential phase, as demonstrated in Fig. 2a. After six h of incubation, all four strains reached the stationary growth phase. Unlike the yellowish colonies of the wild-type and complementary strains, the deleted-mutant colonies were white-colored (Fig. 2b).

### Real-Time qPCR

**Fig. 3:**
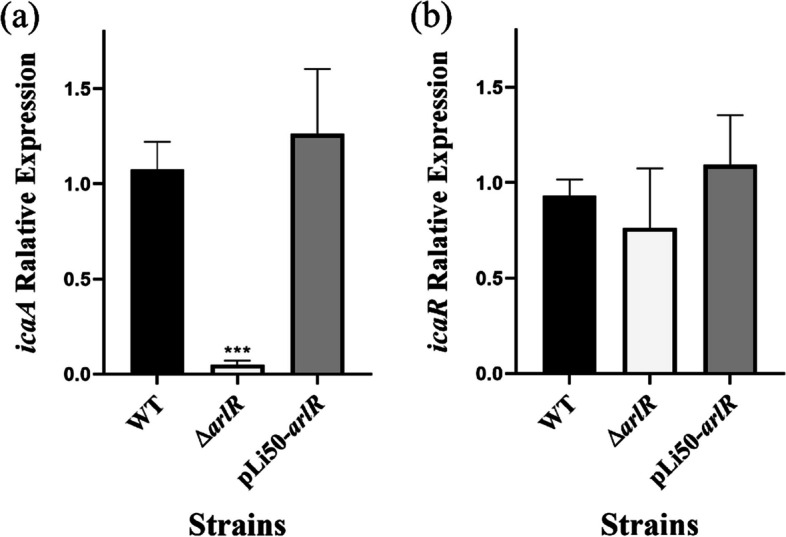
The gene of *arlR* directly affects the expression of the *ica* operon in *S. aureus*. Relative *icaA* and i*caR* transcript levels were evaluated by qRT-PCR. All data represent averages of three separate experiments. The error bars represent the standard deviation (SD) of six biological replicates

As previously mentioned by Yurong Wen et al., *arlR* can bind to the promoter region between *icaR* and *icaADBC*, then regulate the gene expression of *icaADBC* [32]. The impact of *arlR* on *icaADBC* and *icaR* in *S. aureus* biofilm formation and adhesive ability was first executed via the real-time qPCR. Transcript levels of *icaA* and *icaR* were examined at the stationary phase of the wild-type mutant and complement strains. The transcript expression of icaA was reduced almost ten-fold in the complementary strains compared to the wild-type strains (Fig. 3a). Although the transcriptional level of *icaR* did not differ significantly, a minor decrease was seen in the *arlR* mutation but neither in the wild-type nor complementary strains (Fig. 3b). The housekeeping gene 16 S rRNA was used as an endogenous factor to standardize all data. These results demonstrated a reduction in the *icaA* expression of the *arlR* mutation, which was correlated with the adherence and the agglutination. Nevertheless, the *icaR* expression was not affected. The results are consistent with the EMSA results in our previous study, which was activated ArlR recognizes a 20-bp AT-rich inverted repeat [32].

### Adherence and Biofilm Formation Assay

**Fig. 4:**
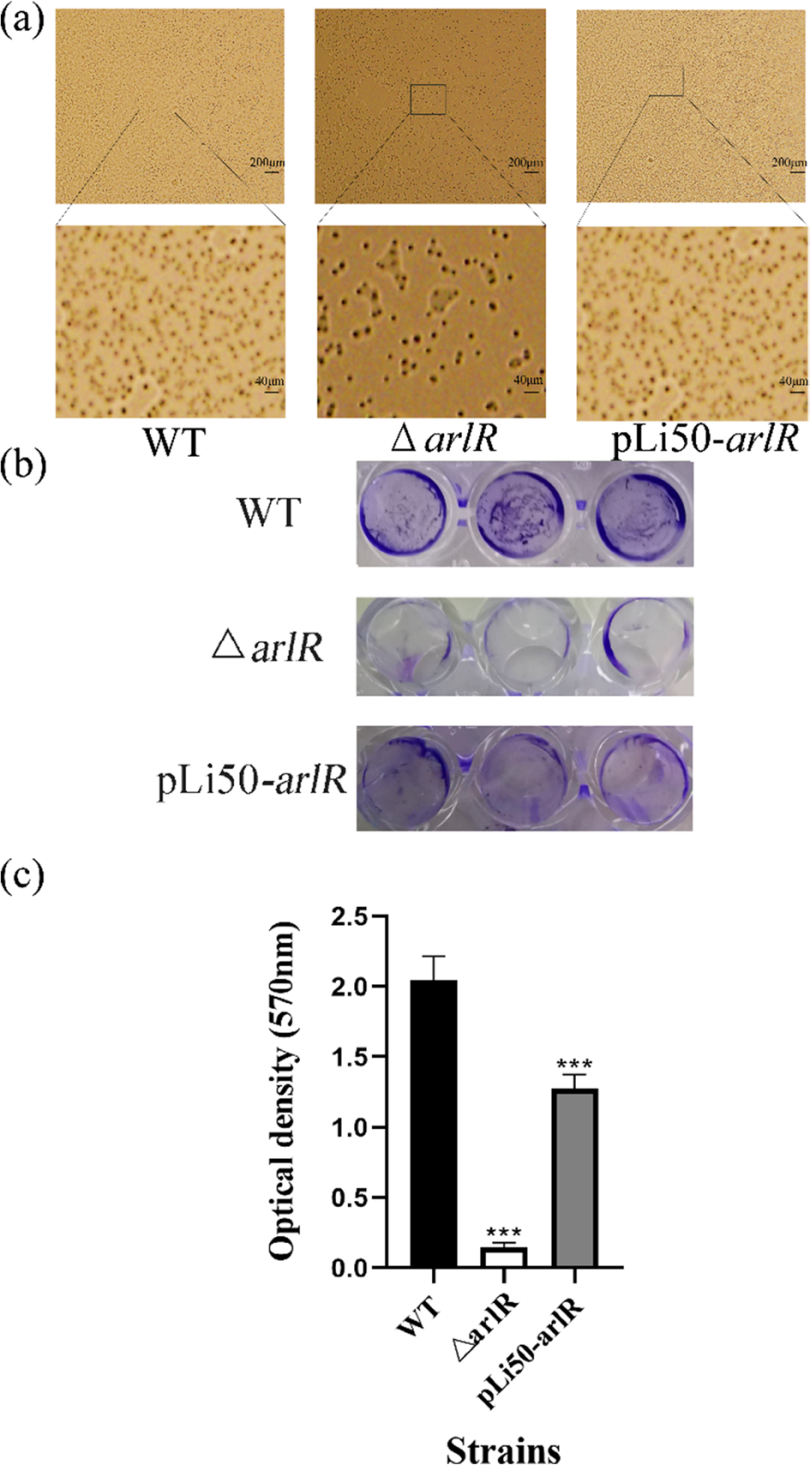
Adherence and biofilm formation capacities of *S. aureus*. **a** Overnight cultures were diluted to 1:100 in 50 mL TSB and grown to the early stationary phase. The adherent cell images were captured using the microscope, with a magnification power of 50× in the upper line and 250× in the lower line. The black boxes in the upper line reflect the regions magnified in the lower line. **b** Overnight cells were diluted by 1:200 with fresh TSB, added into 96-well polystyrene plates, and incubated for 24 h at 37 °C. The biofilms were fixed in 99% methyl alcohol and stained with crystal violet for observation. **c** The bacterial cells were stained with crystal violet, and the quantitative assay of the biofilm formation capacity determined the absorbance at 570 nm. The results of a representative experiment are shown as (*n* = 30; *p* < 0.001), and the error bars represent the standard deviation (SD)

A previous study showed intercellular adherence and accumulation of poly-laminate cell clumps that lead to biofilm formation, which could be measured by crystal violet staining [36]. The attached bacterial cells of wild-type strains showed that the number of adherent cells was significantly higher than the corresponding mutants, with a minor variation between the wild-type and the complementary strains (Fig. 4a). Following incubation of the strains in polystyrene plates, the *arlR* delete mutant appearance (middle panel) had a distinct pattern with the wild-type (upper panel) and complementary strains (lower panel). In contrast to the wild-type strain, the *arlR* delete mutant strain exhibited a negligible affinity for the polystyrene surface, resulting in a minimal adhesion of cell density as measured by 2% crystal violet staining once the biofilm formed on the bacterial surface. The cell density of complementary strains was similar to that of the wild-type (Fig. 4b). Since cell agglutination is an indicator of the strain’s primary attachment and the accumulation for the multilayered cell clusters; therefore, the strains were observed using a spectrophotometer at OD_570_, the wild-type and complementary strains produced several aggregates, and intercellular adhesion significantly decreased in the deleted mutant (Fig. 4c). The *arlR* can reinforce the PIA production, and the PIA is necessary for biofilm and aggregate formation [29, 37]. To find out how *arlR* deleted mutant influences the *icaADBC*, which is essential for PIA biosynthesis, we further study the gene expression levels of these factors.

### Triton X-100-Induced Autolysis in arlR Mutant

**Fig. 5:**
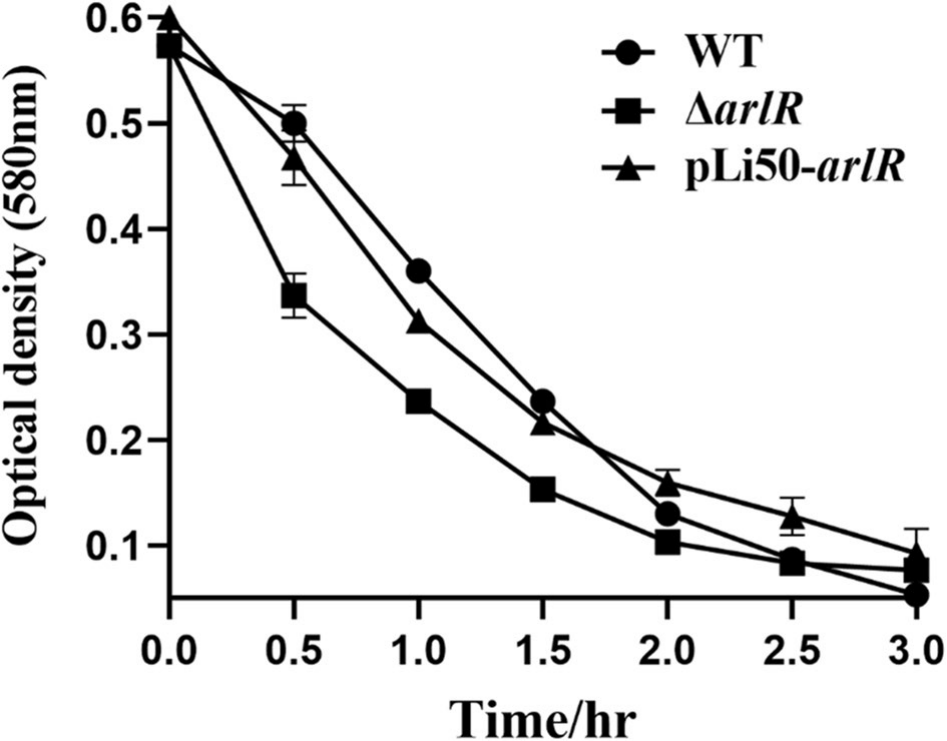
*S. aureus* autolysis of the WT, △*arlR*, and complementary mutant pLi50-*arlR* by 0.01% Triton X-100. The overnight-grown cultures were diluted by 1:100 in TSB containing 1 M NaCl and cultivated at 37 °C till the exponential phase (OD_580_ = 0.7). The experiments were repeated thrice independently

Autolysis plays a vital role in the bacterial cell wall. *S. aureus* has multiple known or putative autolysin genes, including lytM,* lytN*, and *atl*; the expression of autolysins is tightly controlled by several negative autolysis regulators, such as *arlRS* [38]. Aiming to determine whether the mutation leads to an increase in autolysis, we examined the contribution of *arlR* on the cell autolytic activities using Triton X-100. The deleted mutant enhanced the cell lysis level compared to the wild-type strain, while the complementary strain’s lysis behaved similarly to the wild-type one (Fig. 5).

### SEM Images of Biofilms

**Fig. 6:**
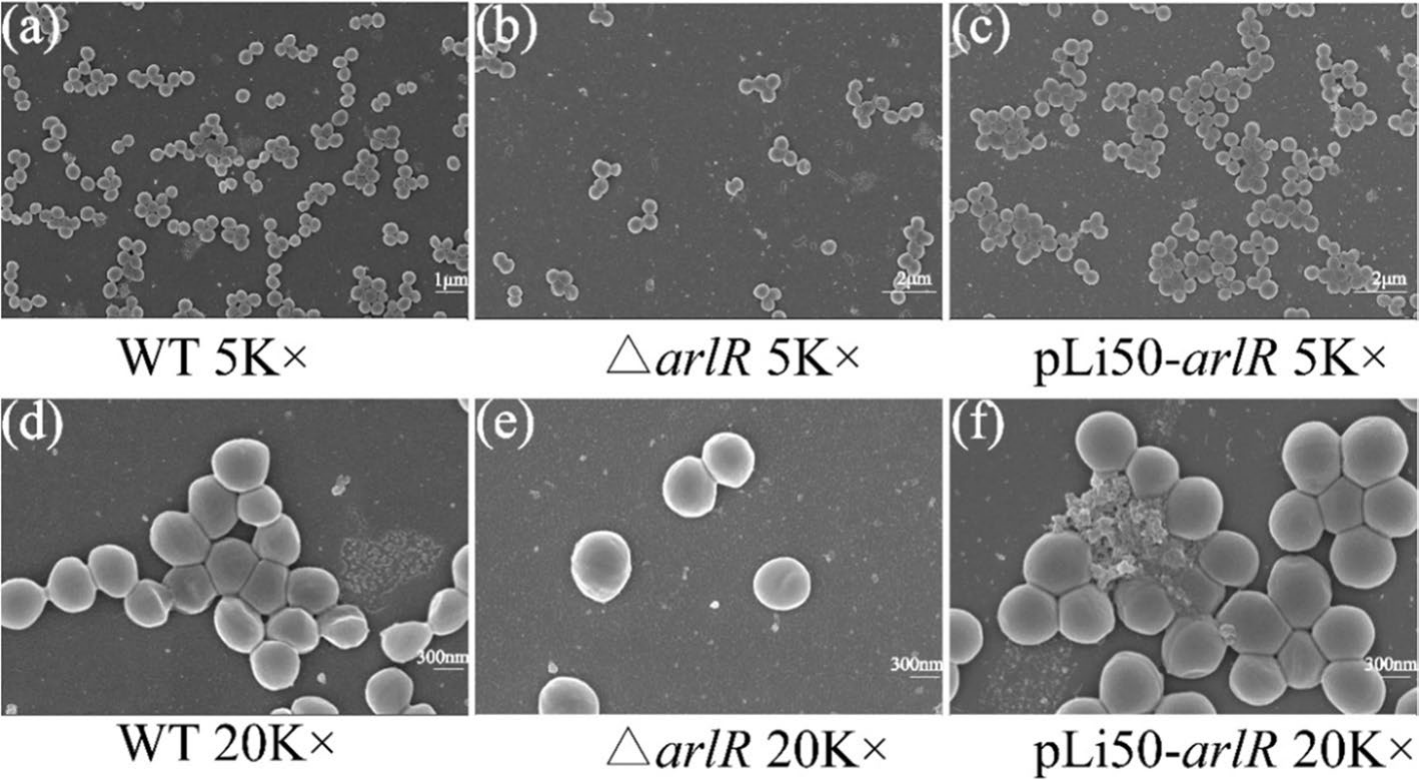
SEM of WT, △*arlR*, and pLi50-*arlR* clumps. *S. aureus* strains were diluted to 1:100 with 1 mL TSB in 24-well plates. After incubation at 37 °C for 24 h, biofilms were observed under an SEM (InLen). Images were taken at different magnifications (×5000, ×20000)

*S. aureus* has been reported to bind with human matrix proteins and form stable clumps in the agglutination process [20]. To further evaluate the clumps-difference between the wild-type, deleted mutant, and complementary strains, the strains were incubated for 24 h at 37 °C to ensure biofilm formation. Scanning electron microscopy (SEM) images indicated a microstructural level of cell morphologies in which the wild-type strains developed a compact, thick biofilm. In contrast, the mutant formed just a few bacterial cell clusters and had clumping defects (Fig. 6a and b). With a 20 K magnification, we observed the cross-links between the wild-type bacterial cells (Fig. 6d and e), which led to an abnormal clump formation. pLi50-*arlR* developed a biofilm similar to wild-type strains (Fig. 6d and f).

## Discussion

As concerns increase regarding antibiotics bacterial resistance and broad-spectrum antibiotics destroying the beneficial microflora, directly targeting the signaling pathways that regulate pathogen virulence emerges as a promising alternative strategy. Biofilm formation has been recognized as a virulence factor attributing the pathogenesis and infections [1, 39]. Despite its clinical significance, little is known about the expression and regulation of *S. aureus* biofilms. Several *S. aureus* genes, such as *ica*, *arlRS*,* and sarA*, have been identified as crucial for biofilm formation. *Ica ADBC*-encoded proteins biosynthesize the PIA, which is essential for *S. aureus* and *Staphylococcus epidermidis* biofilm formation [40, 41]. The *icaADBC* operon plays a vital role in biofilm formation, which is considered a major invasive factor associated with agglutination and pathogenesis [37]. IcaR, located adjacent to the *ica* operon, encodes a transcriptional repressor associated with the *ica* operon expression in *S. aureus* [42].

**Fig. 7:**
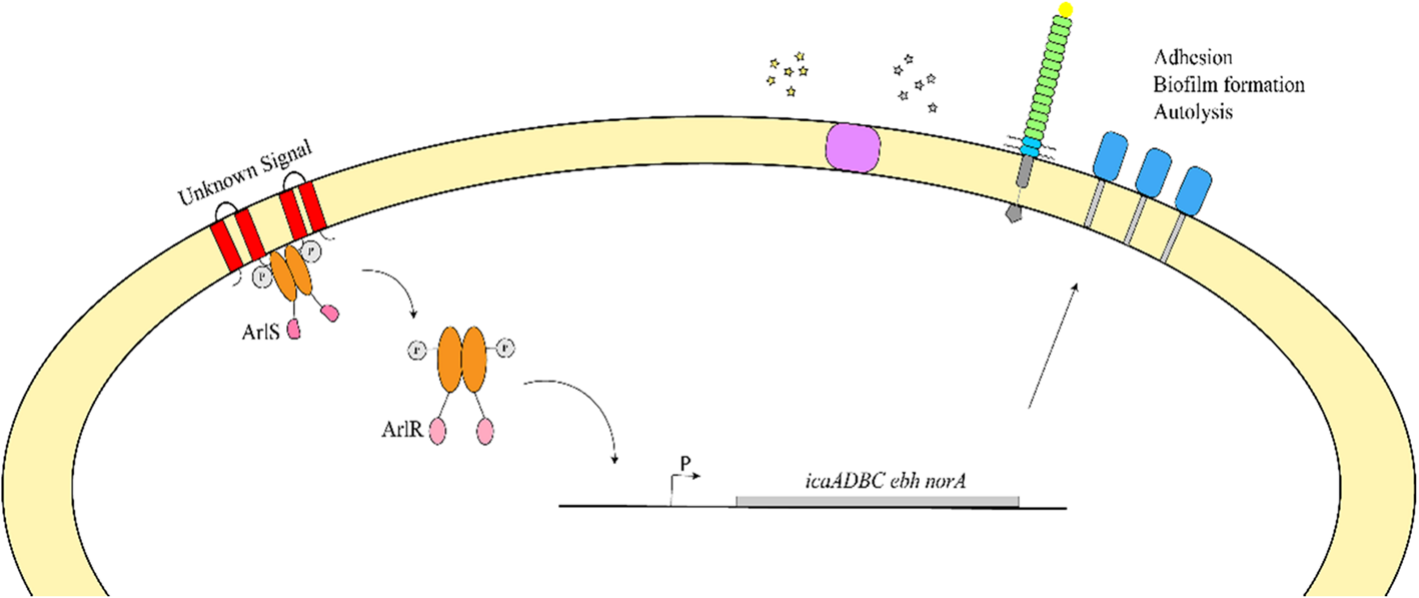
Scheme for ArlRS regulon. ArlR modulates the transcriptional level of *icaADBC* and *icaR*

The response regulator ArlR can bind to the promoter region of the *ica* operon and regulate the expression of *ica* operon and PIA production [14, 43]. Our previous study elucidated the ArlR-binding site located 11 bp upstream of the − 10 sequence, indicating that they are regulated by ArlR directly [32]. This study revealed that the *S. aureus* response-regulatory ArlR influences biofilm formation, agglutination, and autolysis. The result of the biofilm formation assay demonstrated that the deletion of *arlR* has reduced biofilm formation and decreased PIA production (Fig. 7). In *S. epidermidis*,* arlR* could bind with the *icaA* promoter region and the upstream of *icaR*, negatively regulating biofilm formation in an *ica*-dependent manner [44].

The qRT-PCR outcomes revealed that the *icaR* transcriptional level has slightly changed compared to the wild-type strains. Meanwhile, the gene expression of *icaA* has significantly decreased in *arlR* mutants. Triton X-100-induced autolysis showed an increased cell lysis rate in the *arlR* mutant compared to the wild-type and complementary strains. We hypothesize that *arlR* mutations mediate the adherence to polystyrene surfaces and reduce cell clusters by inducing autolysins expression. The SEM images of clumps demonstrated that *arlR* regulated the agglutination on the plastic surfaces. It has been reported that several regulatory factors, including MgrA, Rbf, SigB, TcaR, SrrAB, and Spx, affect *ica* expression, and its regulation is extremely complicated [16, 45].

This study confirmed ArlR’s involvement in the physiological processes of biofilm formation, agglutination, and autolysis in *S. aureus*. However, it did not extensively investigate the molecular-level regulatory mechanism of ArlR, including its response to external signals and the specific genes involved in biofilm formation, agglutination, and autolysis. Although previous studies and this research have demonstrated the direct binding and regulation of icaABC by ArlR, future investigations will focus on elucidating the signaling mechanism of the ArlRS two-component regulatory system and uncovering additional downstream regulatory genes.

## Conclusion

To conclude, the current study discussed the role of the response regulator ArlR in *S. aureus* biofilm formation, agglutination, and autolysis. Biofilm formation is a crucial factor contributing to the pathogeneses and infections caused by *S. aureus*. The expression and regulation of *S. aureus* biofilms are not yet fully understood, but the ica, arlRS, and sarA genes have been identified as key players in biofilm formation. The icaADBC operon, responsible for polysaccharide intercellular adhesin (PIA) synthesis, plays a vital role in *S. aureus* and *S. epidermidis* biofilm formation. The transcriptional repressor IcaR, located adjacent to the ica operon, is associated with the expression of the ica operon in *S. aureus*. The response regulator ArlR has been found to bind to the promoter region of the ica operon, directly regulating its expression and PIA production.

The findings of this study indicate that the deletion of arlR resulted in reduced biofilm formation and decreased PIA production. Additionally, the arlR mutant showed increased autolysis compared to the wild-type and complementary strains. It is hypothesized that arlR mutations mediate adherence to surfaces and reduce cell clusters by inducing the expression of autolysins. Furthermore, arlR was found to regulate agglutination on plastic surfaces. Understanding the mechanisms and regulatory factors involved in biofilm formation is crucial for developing alternative strategies to combat antibiotic resistance and target pathogen virulence directly. Further research is warranted to unravel the complex regulation of biofilm formation and explore the potential of ArlR as a therapeutic target in combating *S. aureus* infections.

## Data Availability

The datasets used and/or analyzed during the current study are available from the corresponding author on reasonable request.

## Abbreviations

*S. aureus*: *Staphylococcus aureus*
ESKAPE: *Enterococcus faecium*, *Staphylococcus aureus*, *Klebsiella. pneumoniae*, *Acinetobacter baumannii*, *Pseudomonas aeruginosa*, and Enterobacter species
MRSA: Methicillin-resistant *Staphylococcus aureus*
MIC: Minimum inhibitory concentration
TCS: Two-component regulatory system
MgrA: Multiple antibiotic resistance regulator A
Ica: Intracellular adhesion
PIA: Polysaccharide intercellular adhesin
USA300: A widely spread community-associated MRSA strain
Rbf: Repressor of biofilm formation
SarX: Sar transcriptional regulatory protein X
SigB: Sigma factor B
SrrAB: Sensor kinase/response regulator system SrrAB
Spx: Global transcriptional regulator Spx
TcaR: Transcriptional regulator of the tca operon
